# Perinatal Mortality in a Northwestern Nigerian City: A Wake up Call

**DOI:** 10.3389/fped.2014.00105

**Published:** 2014-10-08

**Authors:** Mohammed Bello Suleiman, Olugbenga Ayodeji Mokuolu

**Affiliations:** ^1^Department of Pediatrics, Federal Medical Centre, Katsina, Nigeria; ^2^Neonatal Intensive Care Unit, Department of Pediatrics, University of Ilorin Teaching Hospital, Ilorin, Nigeria

**Keywords:** perinatal, mortality, Katsina, Nigeria

## Abstract

**Background:** In Nigeria, of the over 900,000 children under the age of 5 years that die every year, perinatal mortality is responsible for a little over 20%. Previous reports are largely from the southern part of the country. This is the first report of perinatal data from the northwest of Nigeria.

**Methods:** A case control study of perinatal deaths in the three major public hospitals in Katsina metropolis was carried out to determine the pattern of perinatal deaths in the metropolis. Data were collected over a 6 week period on maternal socio-demographic, antenatal, and delivery variables. Data were similarly obtained on neonatal profile and morbidities.

**Results:** There were 143 perinatal deaths (94 stillbirths and 49 early neonatal deaths) out of 1104 live and stillbirths during the study period. The perinatal mortality rate was thus 130 per 1000 births with a stillbirth rate of 85 per 1000 births and an early neonatal mortality rate of 49 per 1000 live births. Stillbirths during the intrapartum period were twice as frequent as macerated stillbirths (2:1). Maternal factors significantly associated with perinatal deaths included chorioamnionitis, ruptured uterus, multiple gestation, medically induced delivery, prolonged labor, unbooked pregnancies, antepartum hemorrhage, and prolonged rupture of membranes. Antepartum hemorrhage was the strongest determinant of perinatal death. Significant neonatal determinants were multiple gestation, severe birth asphyxia, apnea, and necrotizing enterocolitis. Apnea was the strongest neonatal determinant. The majority (83.2%) of perinatal deaths were due to severe perinatal asphyxia (SPA) (54.5%), normally formed macerated stillbirths (20.3%), and immaturity (8.4%).

**Conclusion:** In conclusion, Perinatal Mortality in Katsina metropolis in northwest Nigeria is unacceptably high as we approach the timeline for the millennium development goals. Antepartum hemorrhage and SPA are major determinants.

## Introduction

Reduction of under-five mortality is a key developmental goal of many countries and the millennium development goals (MDGs) (1). To achieve this objective, perinatal mortality, which constitutes a significant proportion of under-five mortality must be reduced. This has become imperative with the increasing prominence of perinatal mortality in places where other causes of under-five mortality are experiencing a downward trend (2). Perinatal deaths result largely from obstetric complications that can be prevented with proper antenatal care and quality neonatal services (3). In designing interventions/strategies to reduce perinatal mortality, it is important to know its magnitude, causes, and determinants in a given locality.

The World Health Organization (WHO) estimated that of the 133 million live births in 2004 worldwide, 3.7 million died in the neonatal period, with 3 million (76%) occurring in the early neonatal period (4). Ninety-eight percent of the deaths took place in the developing world, where 90% of babies were born. In addition, for every early neonatal death (END), an infant was stillborn implying 3 million stillbirths per year. One-third of the stillbirths occurred during delivery from largely preventable causes (3).

The WHO estimated the worldwide perinatal mortality rate (PMR) for the year 2004 as 43 per 1000 births with the stillbirth rate (SBR) of 22 per 1000 births and early neonatal mortality rate (ENMR) of 21 per 1000 births (4). Africa has a PMR of 56 per 1000 births, SBR of 28 per 1000 births, and ENMR 29 of per 1000 births (4). West Africa was second to Central Africa (PMR 69 per 1000 births, SBR 36 per 1000 births, and ENMR 34 per 1000 births) (4).

In Nigeria, of the estimated 5.3 million babies born in the year 2004, there were an estimated 425 000 perinatal deaths with a PMR of 76 per 1000 births, a SBR of 43 per 1000 births, and ENMR of 35 per 1000 births (4). Njokanma et al. (5) reported a PMR of 119.9 per 1000 deliveries in a hospital-based study in Sagamu. Ekure et al. (6) at the Lagos University Teaching Hospital found a hospital-based PMR of 84.8 per 1000 births, while Owa et al. (7) reported a rate of 57.8 per 1000 births in Ilesa, Osun state. Many reports from Nigeria are on perinatal mortality in groups of women with specific complications of pregnancy (8, 9).

Many workers in Nigeria have reported on various determinants and causes of perinatal mortality in their settings (6, 8, 10–19). Determinants of perinatal mortality reported in these studies include maternal illnesses such as diabetes mellitus in pregnancy (10), HIV infection (11, 12), teenage pregnancy (13), cord prolapse (14), pre-eclampsia (15), malpresentation (8), obesity (16), and fetal macrosomia (17). Causes of perinatal mortality reported by these workers include congenital malformations (18), low-birth weight (19), prematurity, and asphyxia (6). There is no previous report from northwestern Nigeria.

A rational way of reducing the under-five mortality is by reducing perinatal deaths. This will be guided by a proper understanding of the causes and determinants of these deaths. The purpose of this study is to identify the magnitude of perinatal deaths, their immediate causes and determinants among babies in Katsina province so that a rational national policy to reduce PMR can be planned and implemented.

## Subjects and Methods

### Study site

The study was conducted at the Federal Medical Centre (FMC), the State General Hospital, and the Turai Umaru Yar’Adua Maternity and Children Hospital (TUYMCH), all located in Katsina metropolis. Katsina is the capital of Katsina State with a population of 318,459 in 2006. The State has a total population of 5,792,578 (provisional 2006 census figure) (20) The FMC provides secondary and tertiary healthcare services in Neonatology and Obstetrics and Gynecology for patients mainly from Katsina metropolis and surrounding Local Government Areas. The General Hospital Katsina and TUYMCH provide secondary healthcare services to the same population. The maternity wings of these hospitals attend to booked, unbooked, and emergency cases. About 27 deliveries are conducted daily with an annual delivery rate of 10,000 in the three hospitals. Deliveries are both vaginal (spontaneous and assisted) and operative.

### Sample size

A total of 143 cases were recruited from July 1st 2011 to August 12th 2011. The minimum number of cases to be recruited for the study from the three centers combined was 119 perinatal deaths. The cases were recruited simultaneously in all three hospitals until the minimum sample size was achieved.

### Ethical clearance

Ethical clearance was obtained from the Ethics Review Committee of the Federal Medical Centre Katsina and State Ministry of Health. The ethical clearance from the State Ministry of Health served as a clearance for the state general hospital and the Turai Umar Musa Yar’Adua women and children hospital.

### Subject recruitment

Cases were recruited from the maternity unit of the three hospitals. Cases are defined as deaths of fetuses and infants from the 28th week of gestational life through the 7th day after birth. They fell into two categories:

1. Stillbirths: fetuses that have died prior to their complete expulsion or extraction from the mother. A weight of 1000 g (corresponding to 28 weeks gestation and crown-heel length of 35 cm) was used as the limit of fetal viability in this study. All stillbirths were further classified into fresh and macerated stillbirths.
  Fresh stillbirths were babies born stillbirth without skin disintegration, skull softening, and lack skin and umbilical cord staining from darkened amniotic fluid. These infants are assumed to have died <12 h prior to delivery.
  Macerated stillbirths on the other hand have disintegrated peeling skin, skull softening, and umbilical cord discoloration by darkened amniotic fluid. Death has usually occurred more than 12 h prior to delivery.
2. All live births that died within 7 days of delivery whether at home or in the hospital (ENDs).

The cases were prospectively recruited, consecutively, and simultaneously, from the three hospitals. Most were recruited from the labor room and maternity theater where most deliveries take place. Those babies that were delivered alive but died were recruited from the place of death, either the SCBU or at home.

Before recruitment, the project was clearly explained to the mother and/or father in a language they understood. One of them signed or used the left thumb to thumb print the informed consent form.

All other babies delivered during the study period were studied as controls. The data obtained on them were compared to that obtained from the cases to determine maternal socio-biologic and neonatal variables associated with perinatal deaths. The total number of babies delivered during the study period was used to calculate PMRs.

### Inclusion criteria

1. All fresh stillbirths delivered in any of the three hospitals during the study period.
2. All macerated stillbirths delivered in any of the three hospitals during the study period.
3. All live births delivered in any of the three hospitals during the study period that died within 7 days of delivery whether at home or in the hospital.

### Exclusion criteria

1. Denial of consent of the caregiver of an eligible subject.
2. Inability to estimate the gestational age of the baby.
3. Failure to trace the baby after delivery (outcome unknown).

### Data collection

Two structured proformas were used for data collection in the study. The first was a questionnaire used to obtain data on all babies delivered in the three study sites during the study period. It had three sections: maternal socio-demographic variables, obstetrics characteristics, and neonatal profile. The second questionnaire was the International Standard Verbal Autopsy Questionnaire for Death of a Child aged under four Weeks developed by the WHO, which was used to determine causes of death in all recruited subjects, i.e., all live births delivered in any of the hospitals during the study period that died in the perinatal period either in the hospital or at home and the stillbirths (fresh and macerated).

### Data analysis

Data from the *pro forma* were entered into a personal computer and analyzed using SPSS version 15. Measures of central tendency and dispersion of quantitative variables, as well as proportions for qualitative variables were determined. Frequency distribution tables of variables were generated. Determinants of perinatal deaths and outcome were cross tabulated and odd ratios determined. Chi-square test (with Yates correction where applicable) and Student’s *t*-test were used to test for association between categorical variables and continuous variables, respectively. The contribution of multiple independent variables on a specific outcome variable was determined using multivariate analysis. For all statistical analysis, *p*-value <0.05 was considered significant.

## Results

### General characteristics of the cases

A total of 143 perinatal deaths were recruited over a 6-week period in the three health facilities. Of these, 80 (55.9%) were delivered at the General Hospital Katsina, 43 (30.1%) in Turai Umar Musa Yar’Adua Maternal and Child Hospital Katsina (TUMYMCH), and 20 (14.0%) at the Federal Medical Centre Katsina. About a third of them, 49 (34.3%), were delivered alive but died during in the early neonatal period; 42 in the hospital, 7 at home. The other 94 were delivered as fresh and macerated stillbirths in a ratio of 2:1 (fresh stillbirths: 63, macerated stillbirths: 31).

### Perinatal mortality rate

There were 1104 live and stillbirths during the study period. The PMR was thus 130 per 1000 live and stillbirths. The SBR was 85 per 1000 deliveries while the ENMR was 49 per 1000 live births. Table 1 below shows the PMR, SBR, and ENMR.

**Table 1:** **Mortality rates of the three study sites**.

	PMR (per 1000 live and stillbirths)	SBR (per 1000 live and stillbirths)	ENMR (per 1000 live births)
Gen Hosp Katsina	145	103	47
TUMYMCH	120	75	48
FMC Katsina	103	51	54
Total	130	85	49

### Determinants of perinatal deaths

A total of 1053 women delivered during the study period in the three health facilities. One of a set of triplets died during the perinatal period. Another 1003 had singleton pregnancies. One hundred and twenty of these were resulted in perinatal deaths. Of the remaining 49 women that had twin gestations, 17 were complicated with perinatal deaths resulting in 22 babies. Thus, the 143 perinatal deaths recruited were products of pregnancies from 138 women.

Socio-demographic variables of the mothers studied as potential determinants of perinatal deaths were age, marital status, educational attainment, occupation, and social class. Women that are grand-multi-parous and those that lack formal education were found to have significantly higher odds of experiencing perinatal death as shown in Table 2.

**Table 2:** **Maternal socio-demographic characteristics association with perinatal death**.

Variable	PD (*n* = 138)	Alive (*n* = 915)	*p*-Value
Low-social class (III–V)	131	855	0.505
Maternal age <18	25	141	0.416
Maternal age ≥35	24	158	0.971
Primiparity	39	237	0.556
Parity ≥5	58	306	0.048
No formal education	87	429	0.000

*PD, perinatal death*.

*Significance = *p*-value <0.05*.

### Maternal antenatal factors

Antenatal factors evaluated to determine their relationship to perinatal deaths were booking status, low-maternal packed cell volume (anemia) at booking (PCV), diabetes mellitus in pregnancy (DM), asthma, sickle cell disease (SCD), human immunodeficiency virus infection (HIV), and pulmonary tuberculosis. Others were antepartum hemorrhage, prolonged rupture of membranes (PROM), pregnancy induced hypertension (PIH), and ruptured uterus. Table 3 summarizes the relationship between these factors and perinatal outcome. Those characteristics with significantly increased odds of perinatal deaths were antepartum hemorrhage, premature rupture of membranes, prolonged rupture of membrane, chorioamnionitis, and PIH.

**Table 3:** **Maternal antenatal variables association with perinatal death**.

Variable	PD (*n* = 138)	Alive (*n* = 915)	*p*-Value
Diabetes mellitus	0	6	0.430
Asthma	3	8	0.165
Sickle cell disease	0	3	0.656
HIV infection	1	4	0.505
Pulm. tuberculosis	0	2	0.755
Multiple gestation	18	32	0.000
APH	35	14	0.000
Abruptio placenta	28	8	0.000
Placenta previa	4	6	0.031
PreROM	17	36	0.000
PROM	19	32	0.000
Chorioamnionitis	7	7	0.001
PIH	35	130	0.001
Unbooked	54	213	0.000

*PD, perinatal death*.

*Significance = *p*-value <0.05*.

### Maternal delivery factors

Table 4 summarizes the relationship between the delivery factors studied as potential determinants of perinatal death and perinatal outcome. Those women who were medically induced to deliver, those that experienced prolonged labor, and those who sustained uterine rupture had significantly higher odds of perinatal death.

**Table 4:** **Maternal delivery characteristics association with perinatal deaths**.

Variable	PD (*n* = 138)	Alive (*n* = 915)	*p*-Value
Medically induced delivery	10	23	0.007
Prolonged labor	32	49	0.000
Ruptured uterus	7	1	0.000

*PD, perinatal death*.

*Significance = *p*-value <0.05*.

### Analysis to exclude confounders of determinants of perinatal death

To exclude confounders, a multiple logistic regression analysis was carried out to evaluate the relative contribution of those factors found to increase risk of perinatal deaths and determine those that remained significant after the analysis. Chorioamnionitis, uterine rupture, multiple gestations, medically induced delivery, prolonged labor, unbooked pregnancies, antepartum hemorrhage, and prolonged rupture of fetal membranes still significantly increased the odds of perinatal deaths (Table 5). The model accounted for 26.9% of the variability in perinatal deaths. Antepartum hemorrhage was the strongest determinant of perinatal death.

**Table 5:** **Maternal risk factors of perinatal deaths**.

	Beta coefficients	*t*	*p*-Value
Primiparity	0.053	1.923	0.055
No maternal education	0.024	0.804	0.422
Multiple gestation	0.125	4.598	0.000
Antepartum hemorrhage	0.319	2.955	0.003
Abruptio placentae	0.088	0.916	0.360
Placenta previa	−0.073	−1.290	0.197
Premature rupture of membranes	−0.024	−0.512	0.608
Prolonged rupture of membranes	0.127	2.684	0.007
Chorioamnionitis	0.083	2.734	0.006
Pregnancy induced hypertension	0.040	1.444	0.149
Unbooked pregnancy	0.071	2.466	0.014
Medically induced labor	0.076	2.778	0.006
Prolonged labor	0.153	5.397	0.000
Ruptured uterus	0.082	2.488	0.013

*Multiple linear regression analysis*.

### Neonatal determinants of perinatal deaths

Neonatal characteristics that were found to increase significantly the odds of perinatal deaths were being a member of a set of twin or triplet gestations, delivery by cesarean section, being a low-birth weight, premature delivery, apgar score at 5 min <7 and resuscitation for more than 5 min as summarized in Table 6.

**Table 6:** **Neonatal characteristics association with perinatal deaths**.

Variable	PD (*n* = 143)	Alive (*n* = 961)	*p*-Value
Twins/triplets	23	78	0.002
Operative delivery	36	101	0.000
**Birth weight category**		
LBW	55	143	0.000
Normal BW	73	724	
Macrosomia	13	93	
**Gestational age**		
Preterm	68	196	0.000
Term	72	746	
Post term	3	19	
**Baby’s classification**		
SGA	4	37	0.644
AGA	128	876	
LGA	9	47	
**5 min apgar score**		
0–3	102	0	0.000
4–6	19	40	
7–10	22	918	
**Duration of resuscitation**		
≥20 min	7	1	0.000
5–19 min	17	43	
<5 min	6	46	

*PD, perinatal death*.

*Significance = *p*-value <0.05*.

Similarly, with the exception of anemia, jaundice, and hypoglycemia, all the morbidities studied in these babies were found to increase the odds of perinatal death significantly as shown in Table 7.

**Table 7:** **Neonatal morbidities association with perinatal deaths**.

Variable	PD (*n* = 49)	Alive (*n* = 961)	*p*-Value
Severe perinatal asphyxia	35	25	0.000
Sepsis	13	36	0.000
Apnea	32	2	0.000
Polycythemia	2	5	0.043
Anemia	1	8	0.369
Respiratory distress	45	69	0.000
Jaundice	5	46	0.102
Hypoglycemia	0	13	0.514
Necrotizing enterocolitis	3	0	0.000
Congenital malformation	5	5	0.000

*PD, perinatal death*.

*Significance = *p-*value <0.05*.

### Analysis to exclude confounders of neonatal determinants of perinatal death

To exclude confounders, a multiple logistic regression analysis was carried out to exclude the relative contribution of morbidities associated with perinatal deaths. Table 8 shows that after the analysis, multiple gestation, operative delivery; severe birth asphyxia, apnea, and necrotizing enterocolitis (NEC) remained significant. The model accounted for 64.4% of perinatal deaths. Apnea was the strongest determinant of perinatal death.

**Table 8:** **Neonatal risk factors of perinatal deaths**.

	Beta coefficients	*t*	*p*-Value
Multiple birth	0.208	3.389	0.001
Premature delivery	0.080	0.852	0.396
Operative delivery	−0.165	−2.599	0.011
5 min Apgar score	0.028	0.318	0.751
Duration of resuscitation	0.028	0.309	0.758
Low-birth weight	−0.040	−0.418	0.677
Severe perinatal asphyxia	0.218	2.371	0.020
Sepsis	0.112	1.667	0.098
Apnea	0.543	6.953	0.000
Polycythemia	0.039	0.626	0.533
Respiratory distress	0.062	0.829	0.409
Necrotizing enterocolitis	0.197	3.164	0.002
Congenital malformations	0.085	1.367	0.175

*Multiple linear regression analysis*.

### Wigglesworth classification of the deaths

**Figure 1:**
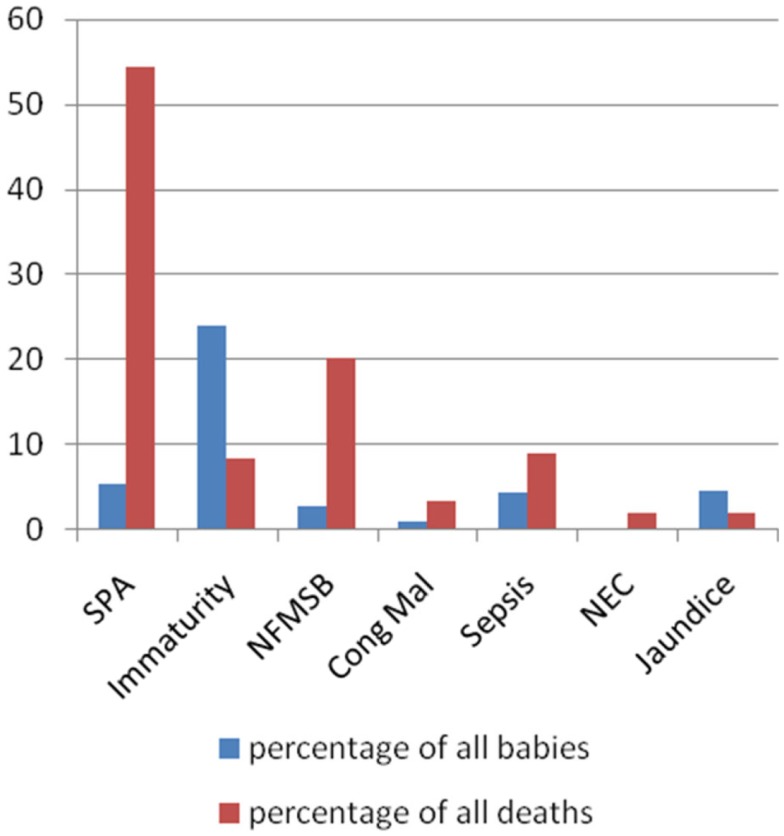
**Wigglesworth classification of perinatal mortality**. SPA, severe perinatal asphyxia; NFMSB, normally formed macerated stillbirth; Cong Mal, congenital malformations; NEC, necrotizing enterocolitis.

Of the 143 perinatal deaths, stillbirths were almost two times as frequent (65.7%) as ENDs (34.3%). Among the stillbirths, fresh stillbirths predominated over macerated stillbirths (2:1). Severe perinatal asphyxia (SPA) was the predominant cause of death (54.5%) (Figure 1). Analysis of cause of death by birth weight shows that SPA was the most important cause of death in all birth weight categories except in the extremely and very low-birth weight babies (ELBW and VLBW) where immaturity and normally formed macerated still births (NFMSB), respectively, were more predominant (Table 9).

**Table 9:** **Wigglesworth classification of perinatal mortality**.

	ELBW	VLBW	LBW	Normal	Macrosomia	Unspecified	Total
SPA	0 (0.0)	4 (18.2)	9 (34.6)	52 (71.2)	11 (84.6)	2 (100.0)	78 (54.5)
Immaturity	6 (85.7)	5 (22.7)	1 (3.8)	–	–	–	12 (8.4)
NFMSB	1 (14.3)	8 (36.4)	7 (26.9)	12 (16.4)	1 (7.7)	–	29 (20.3)
Cong mal	–	2 (9.1)	–	2 (2.7)	1 (7.7)	–	5 (3.5)
Sepsis	–	1 (4.5)	5 (19.2)	7 (9.6)	–	–	13 (9.1)
NEC	–	–	3 (11.5)	–	–	–	3 (2.1)
Jaundice	–	2 (9.1)	1 (3.8)	–	–	–	3 (2.1)
Total	7 (4.9)	22 (15.4)	26 (18.2)	73 (51.0)	13 (9.1)	2 (1.4)	143 (100)

*NFMSB, normally formed macerated stillbirth*.

## Discussion

This study has demonstrated that perinatal mortality remains a significant problem. The PMR in this study may be higher than expected from a community based study because the study centers attend to referrals from primary and other secondary centers. Most normal deliveries are taken in primary and secondary centers, while the complicated ones are referred. This reduces the denominator and thus exaggerates the PMR.

It is higher than the 2004 estimate for Nigeria with a PMR of 76 per 1000 births, a SBR of 43 per 1000 births, and ENMR of 35 per 1000 births (4). The estimate was based on extrapolation from the incomplete vital registrations and studies that were usually hospital-based.

Compared with other reports from Nigeria, the rate is only comparable to that reported by Njokanma et al. (5). It is, however, noteworthy that the Njokanma study took place in Sagamu 20 years before the current study. It is higher than those reported by Ekure et al. (6) and Owa et al. (7). This just re-echoes the poor state of perinatal health in the study population despite various programs by the various tiers of Government and other Non-Governmental Organizations working in the area of perinatal health. As there were no previous studies in the study site to serve as a bench mark, there will be need for other studies in the future to verify whether these programs are succeeding in reducing perinatal deaths.

The study also revealed that the SBR was almost twice the ENMR. This implies that for effective reduction of perinatal mortality, the obstetrician will have an important role to play.

The identified determinants of perinatal deaths in this study (chorioamnionitis, ruptured uterus, medically induced delivery, prolonged labor, unbooked pregnancies, antepartum hemorrhage and PROM, multiple gestation, operative delivery, severe birth asphyxia, apnea, and NEC) are similar to those previously reported in the literature.

Various reasons have been advanced for the association between chorioamnionitis and perinatal deaths. Chorioamnionitis also precipitates preterm labor (21). The incidence of neonatal sepsis is increased in infants born to women with chorioamnionitis (22). All of these were established as important causes of perinatal mortality in this study.

The relationship between ruptured uterus and perinatal mortality has also been reported by previous studies (23–29). It, however, tends to be significantly associated with perinatal death in developing countries where the women tend to present late to hospital when complication sets in (27–29). Hospitals in developing countries are usually under staffed, poorly equipped, overburdened, and usually have non-optimal emergency response. Monitoring of pregnant women in labor is therefore manually performed and poorly executed. The paucity of trained personnel compounds the delays in identification of a ruptured uterus. In developed countries, uterine rupture is usually not a significant cause of perinatal death (24–26).

The risk of perinatal mortality in multiple gestations is more pronounced in developing countries were unsupervised home delivery and late presentation to hospital is common. Even in the hospital, with under equipped and busy labor rooms, monitoring of labor is not very effective in identifying problems early. All these coupled with paucity of skilled personnel and poor emergency response time results in avoidable mortalities. Many other studies have identified multiple gestation as a determinant of perinatal mortality (30–32). The risk is usually higher for the second twin.

Lack of antenatal care results in perinatal deaths probably due to failure of early identification and management of maternal problems that impact negatively on perinatal outcome. Even in advanced economies with sophisticated diagnostic and monitoring equipment, lack of antenatal care categorizes a pregnant woman as a high-risk pregnancy. This obviously becomes more so in developing and under developed countries where the availability of basic equipment and consumables is a major challenge. Many studies have demonstrated the role of lack of antenatal care in poor perinatal outcome (31, 33, 34).

Most of the perinatal deaths that followed antepartum hemorrhage in this study were due to placental abruption and presented as stillbirths (35). This is very important in our locality because of the high rate of lack of antenatal care and late presentation to hospital after complications have arisen. The emergency response time is also quite poor resulting in needless deaths.

Severe perinatal asphyxia was the leading cause of perinatal death in this study (36, 37). It was also the most important cause of death in all birth weight groups except the ELBW and VLBW. The problem of SPA is complicated by late referrals of the mothers, sub-optimal monitoring of labor leading to delayed detection of fetal distress and poor emergency response time when emergency delivery is indicated. There is also lack of awareness and skills of neonatal resuscitation among the delivery attendants. This is despite ongoing efforts by non-governmental organizations to train and retrain the healthcare workers in essential newborn care including neonatal resuscitation.

Neonatal sepsis was an important cause of perinatal mortality in this study. It was responsible for many deaths, especially among VLBW. Sepsis thrives when infection prevention steps are not practiced by delivery attendants. A very important infection prevention strategy is hand washing before touching a patient and in between patients. This was not routinely practiced in any of the delivery rooms where the study was undertaken. This is further compounded by the fact that none of the delivery areas utilized for the study had 24 h tap water supply. Water was stored in containers and used for hand washing and other housekeeping procedures that undoubtedly increased the risk of infection. Another issue noted was none of the facilities has a functional infection control unit. None therefore has an infection control protocol for implementation. There was also no standard operating procedure for identifying and managing babies at risk of infection. All the above could have contributed to infection being an important cause of perinatal death.

Congenital malformations were also important causes of death in this study. Congenital malformations have a spectrum of outcomes. Some are either incompatible with life or associated with very high-case fatalities. Others are relatively benign. Most, however, need highly skilled healthcare professionals for appropriate management. These skilled workers are not readily available. Three of the malformations that eventually died in this study (anencephaly and clinically diagnosed Edward syndrome or trisomy 18), which are normally associated with a very high-case fatality rate. The remaining two, though manageable in facilities with highly skilled workers and facilities, were beyond the capacity of the participating centers to manage.

Jaundice was an important cause of death in this study. Initially identified clinically prior to laboratory confirmation, hyperbilirubinemia was managed by protocol using phototherapy. Only the FMC has the capacity for all the modalities of management of neonatal jaundice. The TUMYMCH can only give phototherapy. The General Hospital does not have facilities for neonatal care. It is therefore not surprising that some babies died as a result of neonatal jaundice because of late presentation and occasional under management.

Necrotizing enterocolitis is an important cause of perinatal mortality. NEC first need to be identified before it is appropriately managed. It needs a highly skilled workforce for appropriate management. It is normally associated with a very high-case fatality even in good centers. Obviously, the level of human resource available in the study centers is under equipped for its optimal management. What might be more practicable will be its prevention. This is, however, a major task with poor infection control protocol practices in the study sites.

The findings of the study highlight the high PMR in the study centers and the roles played by antepartum hemorrhage and SPA, which are surrogate markers for poor antenatal, intrapartum, and postpartum maternal and neonatal care and challenges related to access to care, infection control and safe birth practices.

## Conflict of Interest Statement

The authors declare that the research was conducted in the absence of any commercial or financial relationships that could be construed as a potential conflict of interest.
